# Auranofin and ICG-001 Emerge Synergistic Anti-tumor Effect on Canine Breast Cancer by Inducing Apoptosis via Mitochondrial Pathway

**DOI:** 10.3389/fvets.2021.772687

**Published:** 2021-12-15

**Authors:** Zhaoyan Lin, Zixiang Lin, Ying Zhao, Nan Cheng, Di Zhang, Jiahao Lin, Hong Zhang, Degui Lin

**Affiliations:** ^1^Department of Veterinary Clinical Sciences, College of Veterinary Medicine, China Agricultural University, Beijing, China; ^2^College of Animal Science and Technology, Hainan University, Haikou, China

**Keywords:** auranofin, ICG-001, mitochondrial pathway, ROS, CBC

## Abstract

Canine breast cancer (CBC) is the most common spontaneous tumor in intact female dogs, especially in developing countries. The effective anti-tumor agents or therapies for the clinical treatment of CBC are still in need. Auranofin (AF) is a gold complex that has been attested by FDA for treating human rheumatism, which has been found as a great anti-tumor agent in recent years. ICG-001 is a small molecule inhibitor of Wnt/β-catenin pathway. In the present study, we demonstrated that a combination of AF and ICG-001 could synergistically suppress the proliferation of CBC *in vitro* and *in vivo*. Moreover, the synergistical effect was related with apoptosis caused by mitochondrial damage and ROS production. In conclusion, combination of AF and ICG-001 could synergistically suppress the growth of CBC *in vitro* and *in vivo* by leading apoptosis via mitochondrial signaling pathway and might provide a novel potential choice for the clinical treatment of CBC.

## Introduction

Canine breast cancer (CBC) is the most common spontaneous tumor in intact female dogs, and occurs as the second most common type of tumor in the canine species (1). The surgery is an effective therapy for the most patients bearing with the benign CBC, however, the effective agents or therapies for those who cannot endure surgery or suffer from malignant or metastatic CBC are still being explored. Drug combinations have been widely used and become one of the most leading therapies for the majority of dreadful diseases, such as cancer and infectious diseases (2). The drug synergy may reach more curative effect and reduce the drug dose and side effects. Therefore, the effective combination therapies for cancer treatment are urgently needed.

In the past, mitochondria were exclusively considered as the powerhouses of the cell. However, nowadays it is clear that mitochondrial structure and dysfunction is closely linked to cell death, both in its physiological and pathological occurrence (3–6). There are two major apoptotic pathways: extrinsic and intrinsic pathways responding to different signals in vertebrates, and the intrinsic pathway is also called the mitochondrial pathway owing to the essential involvement of the mitochondria, which is not only the site where antiapoptotic and proapoptotic proteins interact and determine cell fates, but also the origin of signals that initiate the activation of caspases through various mechanisms (5). Caspase family is central components of the machinery responsible for apoptosis, and can be generally divided into three categories: inflammatory caspases (caspase-1, -4, and -5), initiator caspases (caspase-2, -8, -9, and -10), and effector caspases (caspase-3, -6, and -7) (7, 8). Among the initiator caspases, the caspase-9 is involved in the intrinsic pathway, while caspase-8 is involved in the extrinsic pathway; however, caspase-8 also cleaves Bid (a member of Bcl-2 family) and causes cytochrome c release, further amplifies apoptosis via the mitochondrial/intrinsic pathway (9). Mitochondrion is involved in cell apoptosis via regulating the expression of various apoptotic signals related proteins, such as Bcl-2 family, cytochrome c, apoptosis-inducing factor (AIF), etc. Among them, the anti-apoptotic protein Bcl-2 blocks the oligomerization of Bax, and aborts the apoptotic program (10).

In addition, a long-lasting or permanent inner mitochondrial transmembrane potential (ΔΨm) dissipation is often associated with cell death, especially cell apoptosis (4). The basis of ΔΨm is the charge imbalance that results from the generation of an electrochemical gradient across the inner mitochondrial membrane (IMM). During cell death, the IMM cristae will undergo a process of dramatic structural changes as “cristae remodeling,” then cytochrome c will be redistributed from the cristae to the intermembrane space, and then be completely released to the cytosol through apoptotic pores in the outer mitochondrial membrane (OMM) (3, 6).

Reactive oxygen species (ROS) are generally defined as chemically reactive molecules containing oxygen, and produced as a result of cellular metabolism (11, 12). Due to their marked antioxidant capacity, tumor cells often have an altered redox balance to that of their normal counterparts. The redox-imbalanced cancer cells become more vulnerable than normal cells when exogenous ROS-generating agents are triggered. Therefore, ROS manipulation can be a potential target for cancer therapies (13–15). In addition, ROS-dependent anticancer therapies may associate with angiogenesis, apoptosis, oxidative stress, DNA damage, and mitochondrial damage (15).

Auranofin (AF) is a gold complex that has been attested by the FDA for treating human rheumatism. As an inhibitor of thioredoxin receptor (TrxR), AF is considered as an anti-inflammatory drug (16), which may lead to cellular oxidative stress, mitochondrial damage, and pro-inflammatory cell death (17). Moreover, recent research has reported that AF can act as a great anti-tumor agent for multiple tumor types (18–22), and elevate ROS level in cancer cells (23, 24). Besides, researchers have indicated the potential of AF emerging synergistical anti-tumor effect with other drugs (25–27).

ICG-001 is a small molecule inhibitor of Wnt/β-catenin pathway, which has an important impact on initiation and proliferation in various tumor types (28–30). Moreover, ICG-001 is reported to possess anti-tumor ability (31–34), and show the synergistical anti-tumor effect by combining with other agents (32, 35, 36).

## Methods and Materials

### Cell Culture

CIPp (Graduate School of Agricultural and Life Sciences, University of Tokyo, Tokyo, Japan) (37) and CMT-7364 (Veterinary Teaching Hospital, China Agricultural University, Beijing, China) (38) were cultured in DMEM (Life Technologies Inc., Carlsbad, CA) with 10% fetal bovine serum (FBS, Life Technologies Inc., CA), penicillin (100 units/mL), and streptomycin (0.1 mg/mL) (Life Technologies Inc., CA). All cells were cultured at 37°C in an atmosphere containing 5% CO_2_.

### Drug Treatment

AF, ICG-001, and N-acetyl cysteine (NAC) were purchased from MedChemExpress Inc. (Monmouth Junction, NJ). For co-treatment, the cells were incubated with AF and ICG-001 premixed under the dose ratio of 1:10 (2 μM AF, 20 μM ICG-001, 2 μM AF + 20 μM ICG-001) for 24 h; for the experiments regarding the N-acetyl cysteine (NAC) inhibition, the cells were pre-treated with 5 mM NAC dissolved in culture medium for 1 h before drug treatment (17); for the mice co-treatment, mice were treated with AF and ICG-001 premixed under the dose ratio of 1:2 (5 mg/kg AF, 10 mg/kg ICG-001, 5 mg/kg AF + 10 mg/kg ICG-001) for 21 days (39).

### Cell Viability Assay

CCK-8 (TransGen Biotech, Beijing, China) assay was used to measure cell viability. Briefly, 1,000 cells/well were co-incubated with different doses of AF, ICG-001, and the combination in 96-well cell culture plates for 24 h. Later, the value of OD_450_ nm of each well was obtained and recorded with a microplate reader. The experiments were carried out in triplicates and the half inhibition rate (IC_50_) values and the combination index (CI) were calculated to examine the interaction between AF and ICG-001 by Compusyn software (version 1.0, Inc., Paramus, NJ), which integrated the different shape of the curves obtained in calculating the CI value for the evaluation of synergistic effects (40). CI values 1, <1, and >1 indicated an additive effect, synergism, or antagonism, respectively.

### Cell Migration Assay

A wound healing assay was performed to measure cell migration. 3 × 10^5^ cells/well were plated into 6-well cell culture plates. Cells grew to confluence and were scratched with sterile 1,000 μL pipette tips. After scratching, cells were washed twice and co-incubated with drugs for 24 h. The width of wound in each well was recorded by a microscope camera system (CKX41, OLYMPUS, Monolith, Japan), and the migration rate of each well was calculated using the equation: (initial wound width at 0 h—final wound width at 24 h)/width at 0 h × 100% (41, 42).

### Transwell Cell Invasion Assay

The transwell cell invasion assay was performed to measure the capacity of cell invasiveness through extracellular matrix, a process that was commonly found in cancer metastasis (43). There were 24-well inserts (Labgic Co. Ltd., Beijing, China) coated with Matrigel (BD Biosciences, MA) used for cell invasion assays. Briefly, cells were diluted into 1 × 10^4^ cells/mL, resuspended in 100 μL serum-free medium with drugs, and seeded into the upper chamber, while the lower chamber was filled with the medium with 10% FBS. After being cultured at 37°C for 24 h, cells attaching to the bottom side of the upper chamber were stained with 0.1% crystal violet (Solarbio, Beijing, China) and counted under microscope (CKX41, OLYMPUS, Monolith, Japan).

### Colony Formation Assay

Cells were plated on 1,000 cells/well in 6-well plates with drugs (1 μM AF, 10 μM ICG-001, 1 μM AF + 10 μM ICG-001) and further cultured at 37°C, 5% CO_2_ for 10 days. Then the plates were stained with 0.1% crystal violet (Solarbio, Beijing, China), and the colony formation numbers were recorded by photography and counted manually.

### Transcriptome Sequencing Assay

The transcriptome sequencing assay was done by Novogene Co., LTD (Beijing, China). Briefly, 3 × 10^5^ CIPp cells/well were plated into 6-well cell culture plates and treated with drugs for 12 h, and the total RNA was extracted. RNA purity was checked using the NanoPhotometer^®^spectrophotometer (IMPLEN, CA). RNA concentration was measured using Qubit^®^ RNA Assay Kit in Qubit^®^2.0 Flurometer (Life Technologies, CA). RNA integrity was assessed using the RNA Nano 6,000 Assay Kit of the Bioanalyzer 2,100 system (Agilent Technologies, CA). Sequencing libraries were generated using NEBNext^®^ UltraTM RNA Library Prep Kit for Illumina^®^ (NEB, USA) following the manufacturer's recommendations and index codes were added to attribute sequences to each sample. The library fragments were purified with AMPure XP system (Beckman Coulter, Beverly, USA). Then 3 μL USER Enzyme (NEB, USA) was used with size-selected, adaptor-ligated cDNA at 37°C for 15 min followed by 5 min at 95°C before PCR. Then PCR was performed with Phusion High-Fidelity DNA polymerase, Universal PCR primers, and Index (X) Primer. At last, PCR products were purified (AMPure XP system) and library quality was assessed on the Agilent Bioanalyzer 2,100 system. The clustering of the index-coded samples was performed on a cBot Cluster Generation System using TruSeq PE Cluster Kit v3-cBot-HS (Illumia) according to the manufacturer's instructions. After cluster generation, the library preparations were sequenced on an Illumina Hiseq platform and 125 bp/150 bp paired-end reads were generated.

### Transmission Electron Microscope

Briefly, 3 × 10^5^ CIPp cells/well were plated into 6-well cell culture plates and treated with drugs for 24 h, then cells were collected and first fixed with 2.5% glutaraldehyde in phosphate buffer (pH 7.0) for 48 h, washed three times in the phosphate buffer, then postfixed with 1% OsO_4_ in phosphate buffer (pH 7.0) for 1 h and washed three times in the phosphate buffer. Then, the specimen was first dehydrated by a graded series of ethanol (30, 50, 70, 80, 90, 95, and 100%) for about 15–20 min at each step, and transferred to absolute acetone for 20 min. After that, the specimen was placed in a 1:1 mixture of absolute acetone and the final Spurr resin mixture for 1 h at room temperature, then transferred to 1:3 mixture of absolute acetone and the final resin mixture for 3 h, and to a final Spurr resin mixture overnight. Next, the specimen was placed in capsules containing embedding medium and heated at 70°C for about 9 h. Finally, the specimen sections were stained by uranyl acetate and alkaline lead citrate for 15 min, respectively, and observed in TEM (H-7650, Hitachi Ltd., Tokyo, Japan).

### Mitochondrial Membrane Potential Assay

There were 3 × 10^5^ cells/well plated into 6-well cell culture plates and treated with drugs for 24 h. Later, ΔΨm in cells was detected by following the instruction of mitochondrial membrane potential assay kit with JC-1 (C2006, Beyotime Biotechnology, Beijing, China). Then images were captured with NIS-Element Viewer (version 5.21.0, Nikon Instruments Inc., Shanghai, China).

### Flow Cytometry Assay

There were 3 × 10^5^ cells/well plated into 6-well cell culture plates and treated with drugs for 24 h, then cells were collected and washed with PBS. JC-1 agent was added following the instruction of mitochondrial membrane potential assay kit with JC-1 (C2006, Beyotime Biotechnology, Beijing, China). After that, samples were collected and analyzed on a FACS Calibur flow cytometer (BD Biosciences, USA), and data were analyzed with Flow Jo software (Version 7.6.1, USA).

### ROS Assay

Reactive Oxygen Species Assay Kit (Solarbio, Beijing, China) was used to detect the ROS level after drug application following the instructions. Briefly, 1,000 cells/well were co-incubated with drugs in 96-well cell culture plates for 24 h. Later, the value of each well at OD_525_ nm was obtained and recorded with a fluorescence microplate instrument.

### Western Blot Assay

Protein was extracted from harvested cells and quantified through bicinchoninic acid (BCA) analysis (Life Technologies Inc., CA). Equal amounts of protein were loaded. The following primary antibodies were used for Western blot analysis: β-Actin (66009-1- Ig, Proteintech, Rosemont, IL, 1:2000), α-Tubulin (66031-1-Ig, Proteintech, Rosemont, IL, 1:2000), Bcl-2 (12789-1-AP, Proteintech, Wuhan, China, 1:1000), Bax (50599-2-lg, Proteintech, Wuhan, China, 1:2000), cleaved-Caspase-3 (9661T, Cell Signaling Technology, Danvers, MA, 1:1000), cleaved-Caspase-8 (8592T, Cell Signaling Technology, Danvers, MA, 1:1000), cleaved-Caspase-9 (9509T, Cell Signaling Technology, Danvers, MA, 1:1000). After incubated with primary antibodies and washed 5 times, the membranes were incubated with secondary antibodies conjugated with horseradish peroxidase (HRP) anti-mouse/rabbit IgG (SA00001–9, SA00001–1, Proteintech, Rosemont, IL, 1:5000), washed 5 times, and exposed under chemiluminescent imaging analysis system (Tanon 5200, China). Densitometry analysis was done by Image J (Version 1.50i, National Institutes of Health, USA).

### TUNEL Assay

Deoxynucleotidyl Transferase-Mediated dUTP Nick End Labeling (TUNEL) assay was performed to measure cell apoptosis. Briefly, 5 × 10^4^ cells were plated on cell culture slides in 12-well plates until ~70% confluent. Cells were then treated with drugs for 24 h. After fixation in 4% paraformaldehyde for 1 h, samples were stained with TUNEL reagent (TransGen, Beijing, China) following the manufacturer's instructions. Then images were captured with a Nikon confocal microscope.

### Tumor Xenograft Mouse Model

The animal study was reviewed and approved by the Animal Ethics Committee of the China Agricultural University (approval code AW10901202-2-1), according to the guidelines for Laboratory Animal Use and Care from the Chinese Center for Disease Control and Prevention and the Rules for Medical Laboratory Animals (1998) from the Chinese Ministry of Health, under protocol CAU20151001-1.

To establish a tumor xenograft mouse model, 1 × 10^6^ CIPp cells were resuspended in 150 μL PBS and injected subcutaneously into the left mammary fat pad of 5-week-old female null-balb/c mice (Vital River, Beijing, China). When the tumor length reached about 5 mm, mice were then randomly assigned into 4 groups (*n* = 6) and treated with drugs or vehicle by intraperitoneal injection daily for another 21 days. Tumor sizes were measured with a digital caliper every 3 days. The volume of the tumor size was calculated as follows: V = L × W^2^/2, of which W corresponds to the width and L to the length. After treatment, the lungs, livers, kidneys, spleens, and hearts were harvested to perform pathological section with HE staining, and the tumors were collected to perform the TUNEL assay.

### Statistical Analysis

Statistical analysis was performed by using GraphPad Prism5 software (version 5, GraphPad Software Inc., San Diego, CA). A two-tailed unpaired *t*-test with Welch's correction was applied when the variances of two groups were proved equal by the *F* test, and *p*-values of 0.05 or less were the threshold for statistical significance.

## Results

### Combination of AF and ICG-001 Synergistically Suppresses Cell Viability of CBC

**Figure 1:**
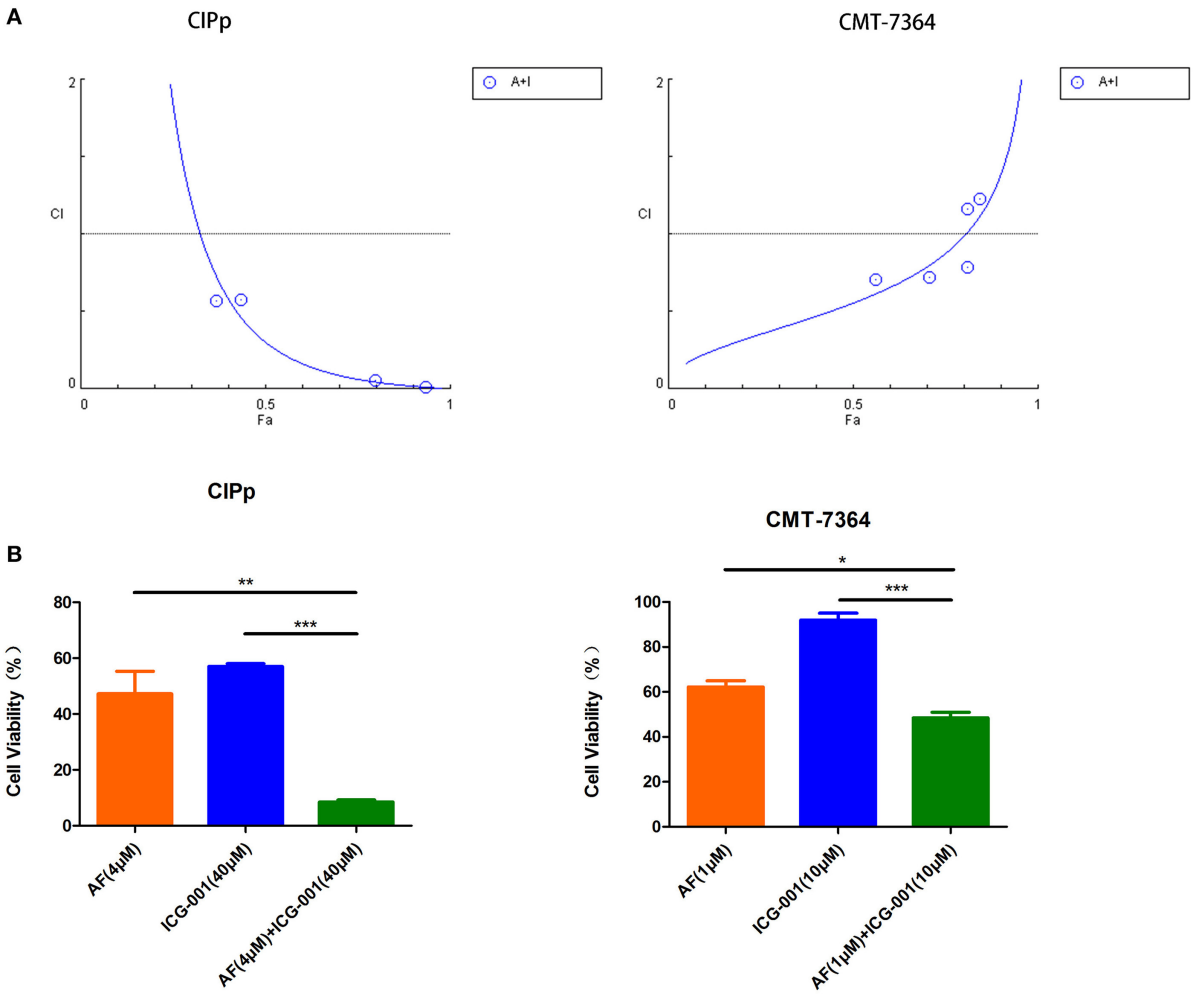
Combination of AF and ICG-001 Synergistically Suppresses Cell Viability of CBC. **(A)** The combination index (CI) of the combination of AF and ICG-001 of CIPp and CMT-7364 cell lines. Fa, fraction affected. **(B)** The cell viability of CIPp and CMT-7364 cell lines after drug treatment. Data were representative of three independent experiments. *p*-values: *p < 0.05; **p < 0.01; ***p < 0.001.

To explore the anti-tumor effect of AF and ICG-001 on CBC *in vitro*, we performed the cell viability assay to detect cell viability after drug treatment. The results showed that AF and ICG-001 could both suppress cell proliferation of CIPp and CMT-7364 cell lines, the IC_50_ value of AF on CIPp and CMT-7364 were 2.1376 and 1.2699 μM, respectively; the IC_50_ value of ICG-001 on CIPp and CMT-7364 were 72.6354 and 79.1305 μM, respectively (Table 1). Meanwhile, the combination index (CI) was calculated to describe the combination effect of AF and ICG-001 on CBC. The CI value was <1 in both cell lines (Figure 1A), for example, the CI value at 70% cell inhibited were 0.08620 for CIPp cells and 0.78854 for CMT-7364 cells, respectively. Then, we confirmed this synergism of combination therapy by analyzing cell viability assay (Figure 1B). Those results indicated AF and ICG-001 could synergistically suppress the cell proliferation of CBC *in vitro*.

**Table 1:** Dose-effect relationships of single drugs and combination in CBC cell lines.

**Cell line**	**Single drugs** **and combination**	**Parameters**			**CI value at**				**DRI value at**			
		**Dm**	***m***	***r***	**ED50**	**ED70**	**ED90**	**ED95**	**ED25**	**ED50**	**ED75**	**ED95**
CIPp	AF	2.13764	0.37677	0.90901					0.59835	4.30202	30.9305	850.730
	ICG-001	72.6354	0.61159	0.91772					6.22824	14.6180	34.3091	143.860
	AF+ICG-001	5.46580	1.16480	0.98009	0.30086	0.08620	0.01692	0.00813				
CMT-7364	AF	1.26990	0.91715	0.94630					3.18730	2.09064	1.37131	0.67519
	ICG-001	79.1305	1.12886	0.98962					24.8635	13.0273	6.82567	2.30417
	AF+ICG-001	6.68164	0.67834	0.97965	0.55508	0.78854	1.39137	1.91505				

*Dm, median-effect dose (concentration that inhibits cell growth by 50%); m, shape of the dose effect; r, linear correlation of the median-effect plot; CI, combination index; DRI, dose-reduction index*.

### Combination of AF and ICG-001 Suppresses Cell Migration, Cell Invasion, and Colony Formation of CBC

**Figure 2:**
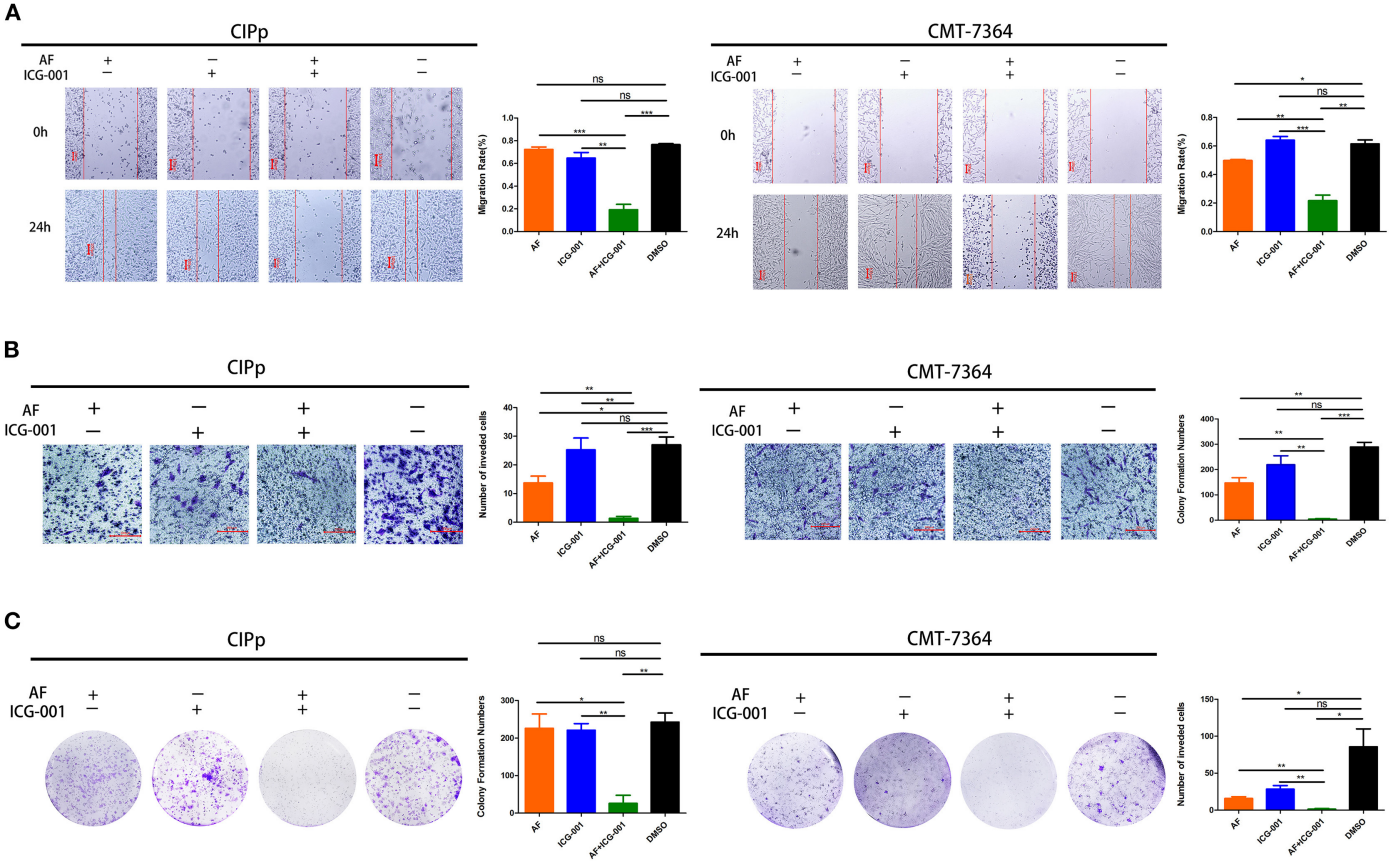
Combination of AF and ICG-001 suppresses cell migration **(A)**, cell invasion **(B)**, and colony formation **(C)** of CIPp and CMT-7364 cell lines. Bar, 100 μm **(A)** and 200 μm **(B)**. Data were representative of three independent experiments. *p*-values: *p < 0.05; **p < 0.01; ***p < 0.001; ns, non-significant.

In order to further investigate the influence of AF and ICG-001 treatment on cell migration, invasion, and colony formation capability of CBC *in vitro*, we conducted cell migration assay, transwell invasion assay, and colony formation assay. As the results indicated, the ability of migration (Figure 2A), invasion (Figure 2B), and colony formation (Figure 2C) of CIPp and CMT-7364 cells was significantly greater inhibited in the combination group than those in the control group or the individual groups.

### Combination of AF and ICG-001 Significantly Regulates the Expression of Redox Related Genes

**Figure 3:**
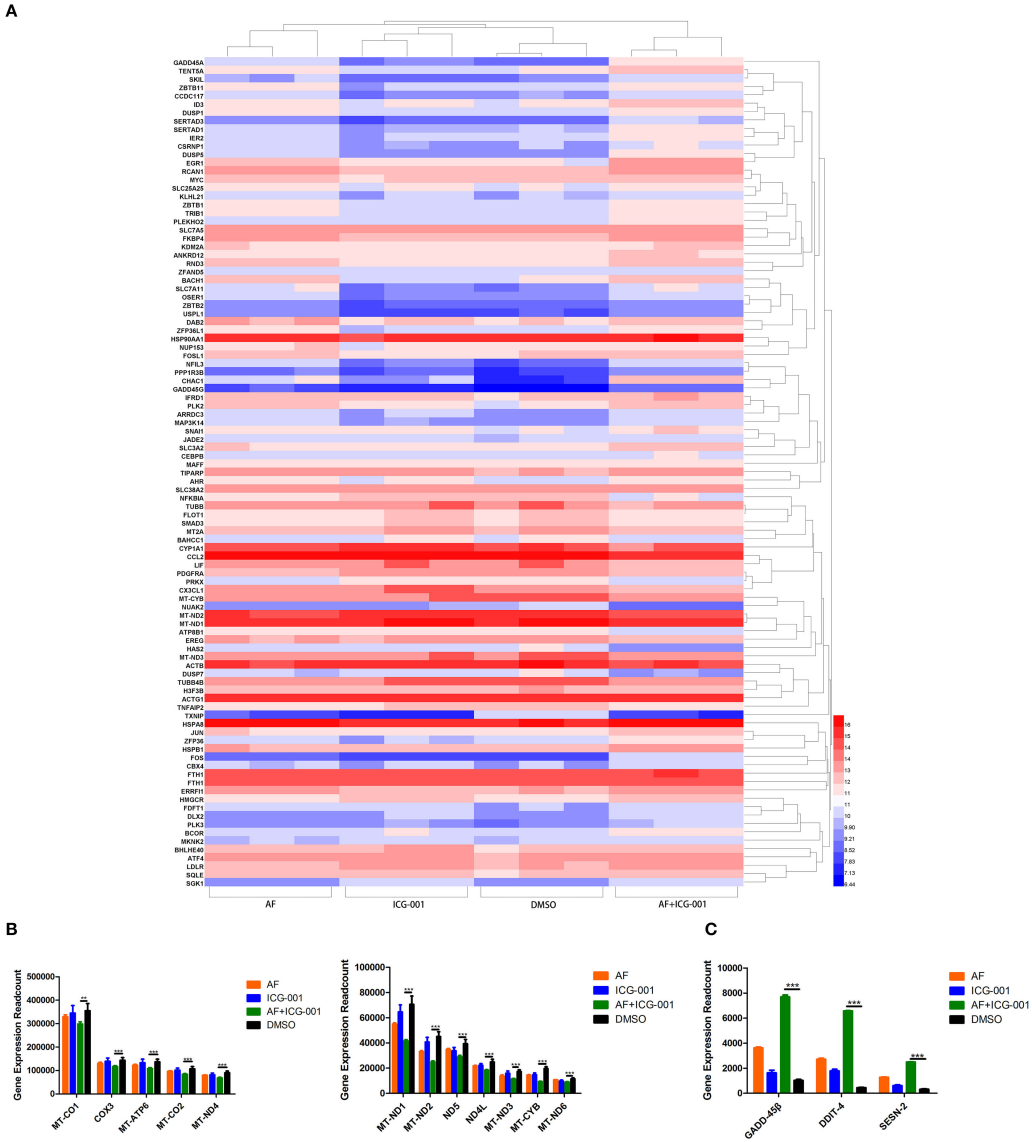
Combination of AF and ICG-001 significantly regulates the expression of redox related genes. **(A)** The heatmap of 100 most differentially expressed genes of CIPp cells. **(B)** Read count of the significantly changed redox related genes. **(C)** Read count of some apoptosis-related genes. Data were representative of three independent experiments. *p*-values: *p < 0.05; **p < 0.01; ***p < 0.001.

For the purpose of exploring the underlying mechanism of the combination therapy, we performed a transcriptome sequencing of CIPp cells. As the results showed that in total 33,625 genes were detected, and then we analyzed the 100 most differentially expressed genes of CIPp cells in the combination group than those in the control group (Figure 3A). Among those differentially expressed genes, we noticed that multiple redox related genes were significantly up-regulated in the combination group compared with those in the control group (Figure 3B). Meanwhile, several genes related with cell apoptosis, such as Growth Arrest and DNA-Damage-Inducible-45Beta (GADD-45β), DNA-Damage-Inducible-Transcript-4 (DDIT-4), sestrin-2 (SESN-2), were also significantly up-regulated in the combination group (Figure 3C).

### Combination of AF and ICG-001 Causes Mitochondrial Damage

**Figure 4:**
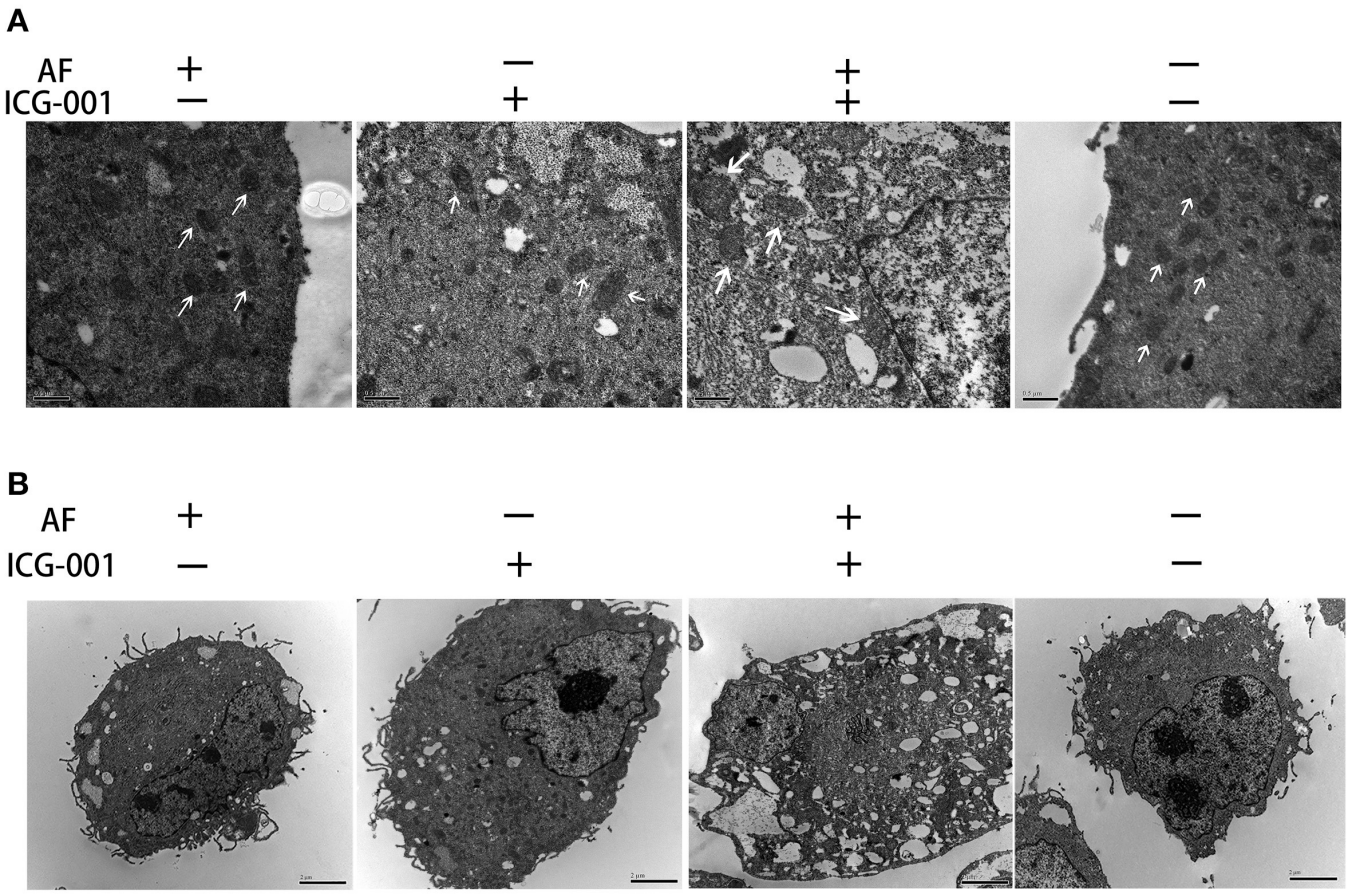
Combination of AF and ICG-001 causes mitochondrial damage. **(A)** The TEM images of mitochondrion. Arrow: mitochondrion. Bar, 0.5 μm. **(B)** The TEM images of cell morphology. Bar, 2 μm.

To visually explore the influence of combination AF and ICG-001 on the mitochondria of CBC, we arranged a TEM assay. As the results showed, the mitochondria of CIPp cells in the combination group presented swelling, vacuolization, and membrane disruption, but not in the individual groups and the control group (Figure 4A). Meanwhile, the cell morphology also changed after the combination treatment, such as loose cytoplasm (Figure 4B) and numerous autophagosome (Supplementary Figure S1).

### Combination of AF and ICG-001 Influences Mitochondrial Membrane Potential and Induces ROS Production

**Figure 5:**
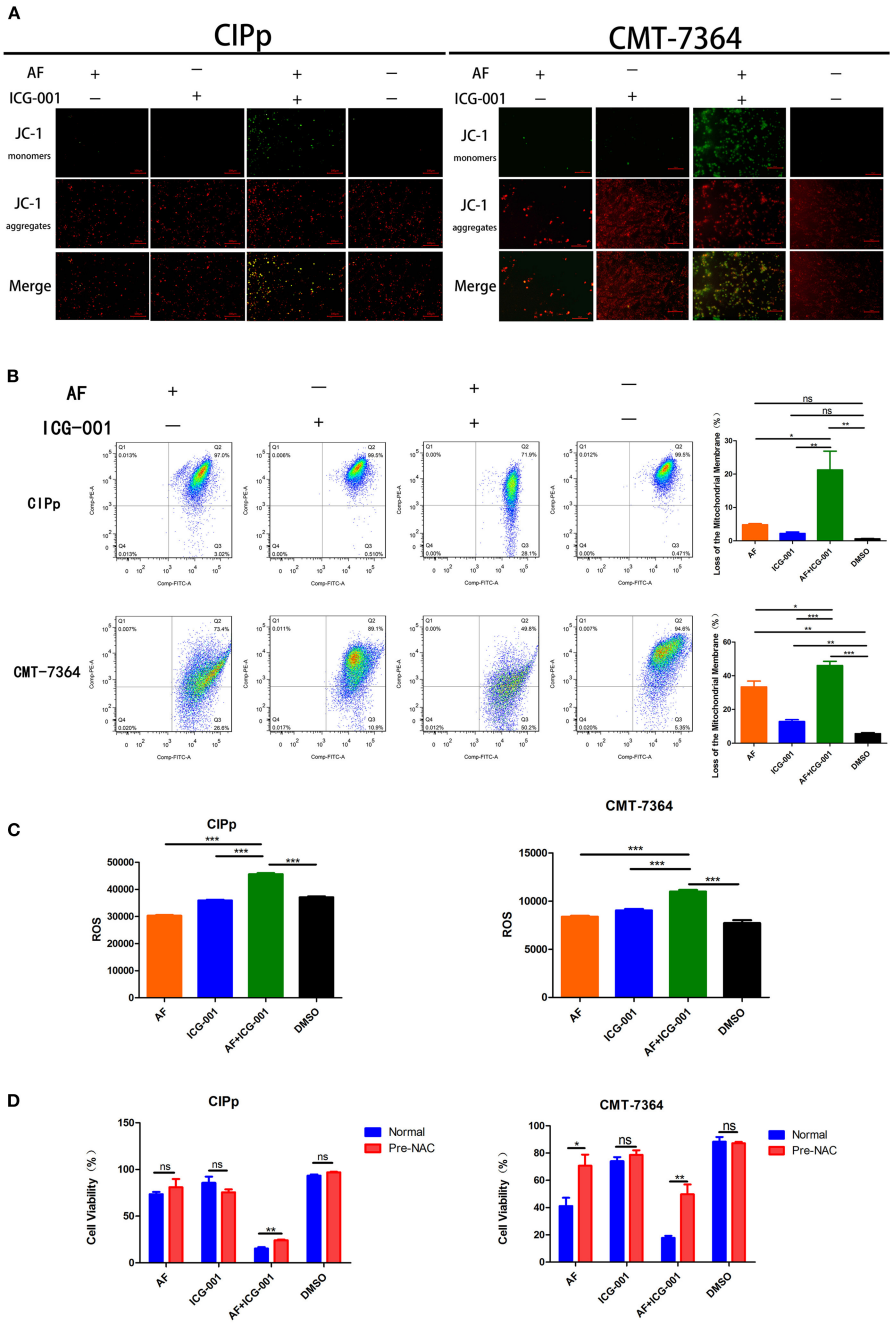
Combination of AF and ICG-001 influences the mitochondrial membrane potential and induces ROS production. **(A)** Detection of mitochondrial membrane potential (MMP, ΔΨm) of CIPp and CMT-7364 cell line by using JC-1 agent. Bar, 100 μm. **(B)** The ΔΨm of CIPp and CMT-7364 cells detected by the flow cytometry assay. **(C)** ROS level of CIPp and CMT-7364 cells after drug treatment. **(D)** Cell viability of CIPp and CMT-7364 cell lines after drug treatment (4 μM AF, 40 μM ICG-001, 4 μM AF+40 μM ICG-001) with or without pre-NAC application. Data were representative of three independent experiments. *p*-values: *p < 0.05; **p < 0.01; ***p < 0.001; ns, non-significant.

To confirm the results of the transcriptome sequencing and TEM, we then detected the mitochondrial membrane potential (MMP, ΔΨm) of CIPp and CMT-7364 cells after drug treatment by using JC-1 agent. When ΔΨm is high (which occurs in normal cells), JC-1 will accumulate in mitochondrial matrix and format aggregates, which shows red color under fluorescence microscope; while when ΔΨm is low, JC-1 cannot accumulate and will exist as monomers, which presents green color under fluorescence microscope. As the results showed, the number of cells with low ΔΨm in combination group was significantly larger than that in the control group and individual groups (Figure 5A). In order to perform quantitative analysis, a flow cytometry assay was done to count the low ΔΨm cells, and the regulatory trend of the results was the same with those of cyto-fluorescence assay (Figure 5B).

Since ROS is closely related to mitochondrion, we supposed that the anti-tumor effect of combination of AF and ICG-001 might relate to excessive production of ROS. Then, an ROS assay was developed, and the results indicated that the ROS level of CIPp and CMT-7364 cells in the combination group was significantly higher than that in the control group or individual groups (Figure 5C). To confirm these results, we employed NAC, a thiol-reducing antioxidant, to examine the role of intracellular ROS in this combined treatment. As the results showed, NAC significantly rescued cell viability of CBC in the combination group, but not in other groups except that in the AF treating group of CMT-7364 (Figure 5D).

### Combination of AF and ICG-001 Induces Cell Apoptosis

**Figure 6:**
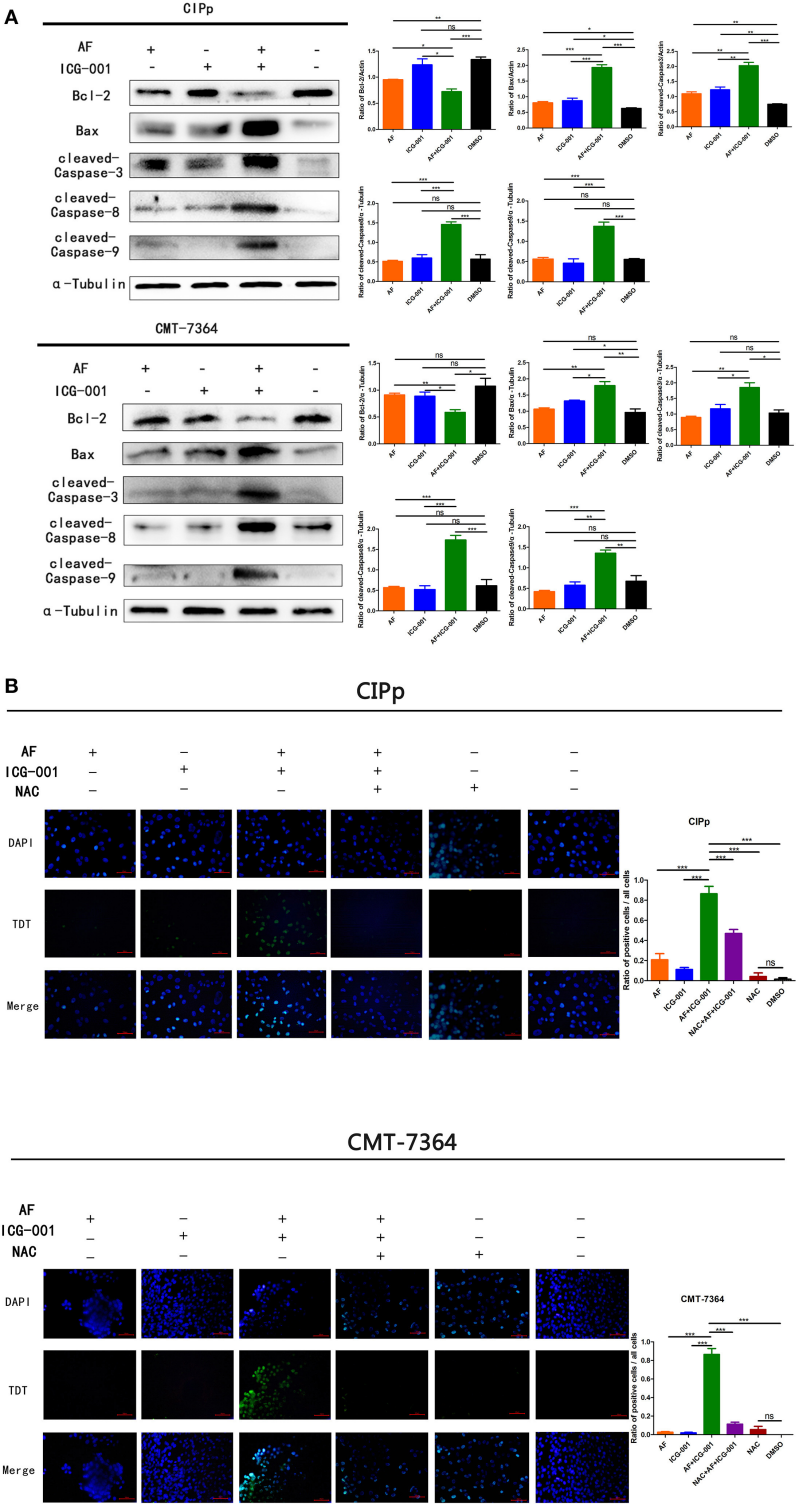
The combination of AF and ICG-001 induces cell apoptosis. Western blot **(A)** and TUNEL **(B)** results indicated that the combination of AF and ICG-001 induced cell apoptosis of CIPp and CMT-7364 cell lines. Data were representative of three independent experiments. *p*-values: *p < 0.05; **p < 0.01; ***p < 0.001; ns, non-significant.

Mitochondrial damage, low ΔΨm, and ROS production are closely related to cell apoptosis. Therefore, we assessed whether AF and ICG-001 treatment would induce cell apoptosis on both CIPp and CMT-7364 cell lines. The results showed that the expression of Bcl-2, an apoptosis regulator, was significantly decreased in the combination group compared with that in other groups, and the expression level of Bax, an apoptosis promoting protein, was significantly raised in the combination group, further, cleaved-Caspase-8, cleaved-Caspse-9, and cleaved-Caspase-3 expression level in the combination group were significantly higher than those in other groups (Figure 6A). These results indicated that a combination of AF and ICG-001 actually caused cell apoptosis depending on the mitochondrial pathway in CIPp and CMT-7364 cell lines.

To further verify the apoptotic effect of this combination therapy, a TUNEL assay was performed for CIPp and CMT-7364 cell lines. As a result, the combination group showed significantly more apoptotic cells than individual groups and the control group. Meanwhile, the pre-NAC treated combination group showed fewer apoptotic cells than the normal combination group (Figure 6B), indicating that the apoptosis induced by combination therapy was rescued by NAC, a ROS inhibitor. Therefore, we inferred that the apoptotic effect induced by the combination therapy was related with ROS production.

### Combination of AF and ICG-001 Suppresses CBC Growth *in vivo*

**Figure 7:**
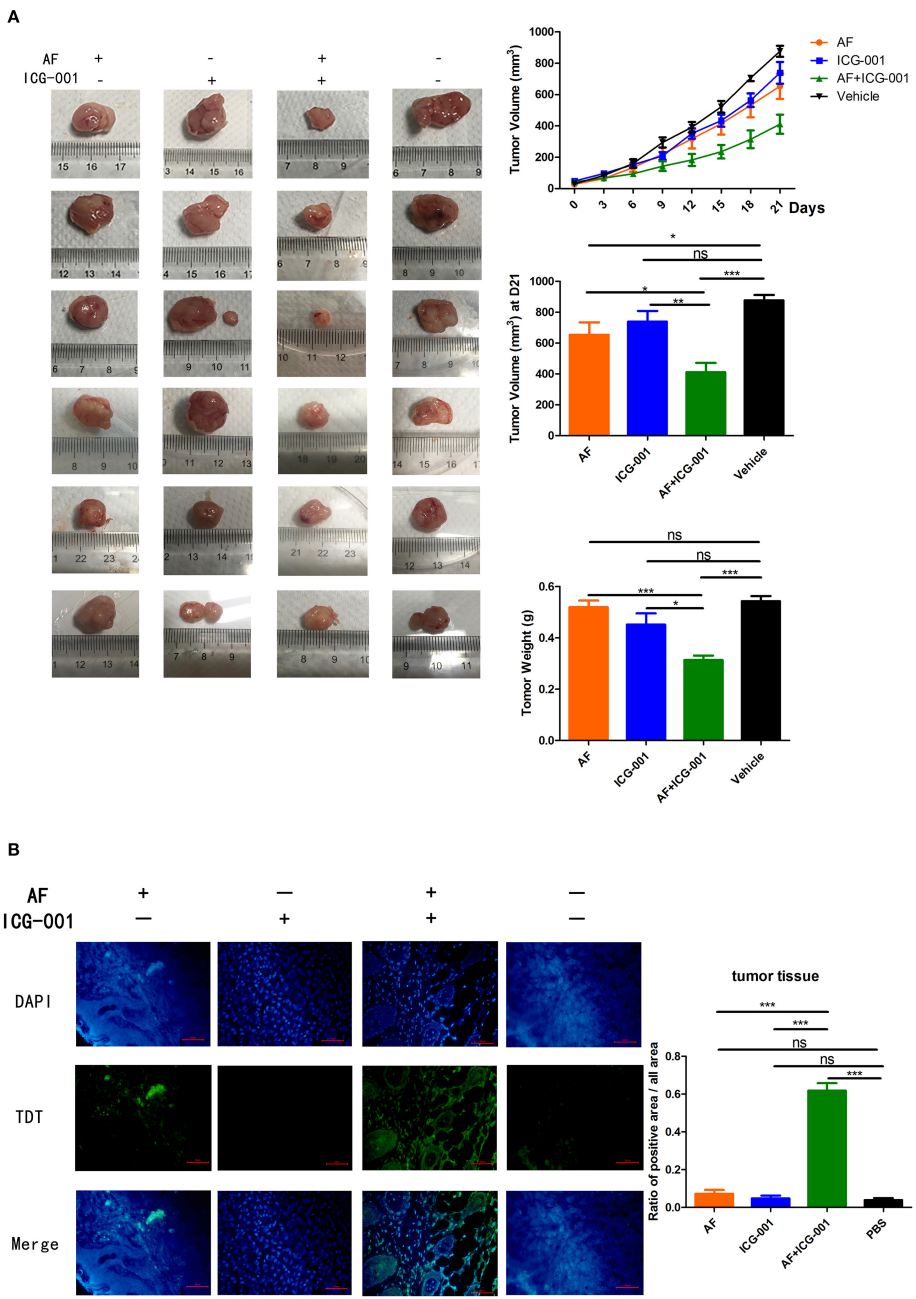
Combination of AF and ICG-001 suppresses CBC growth *in vivo*. CIPp cells were injected subcutaneously into the left mammary fat pad of 5-week-old female null-balb/c mice, *n* = 6. **(A)** The tumor volume during treatment and the tumor weight by the end of the treatment. **(B)** TUNEL results of the tumor tissues at the end of the treatment. Data were representative of three independent experiments. *p*-values: *p < 0.05; **p < 0.01; ***p < 0.001; ns, non-significant.

In order to explore the anti-tumor effect of this combination therapy *in vivo*, the mice were transplanted with CIPp cells to build a tumor xenograft mouse model and then divided into four groups (*n* = 6). After the 21-days of treatment, although applying AF or ICG-001 alone could inhibit the tumor growth to a certain extent compared with the control group, the tumor volume and tumor weight of mice in the combination group were significantly smaller and lighter than those in individual groups and the control group (Figure 7A). The results showed synergistical anti-tumor effect of AF and ICG-001 on mice bearing with xenograft tumor.

Meanwhile, in order to evaluate the drug toxicity, mice body weight was measured, and the lungs, livers, kidneys, spleens, and hearts were harvested to perform pathological section with HE staining at the end of treatment. The results showed there were no significant change of mice body weight among groups during treatment (Supplementary Figure S2A), and there were also non-specific visceral changes in the pathological sections among groups (Supplementary Figure S2B). Therefore, those results indicated that there was no obvious side effect *in vivo* after these drug treatments.

To further verify the apoptosis-inducing effect of this combination therapy, a TUNEL assay was also done with the tumor tissues harvested at the end of the treatment, and the results showed the combination of AF and ICG-001 induced cell apoptosis of CBC *in vivo* (Figure 7B).

**Figure 8:**
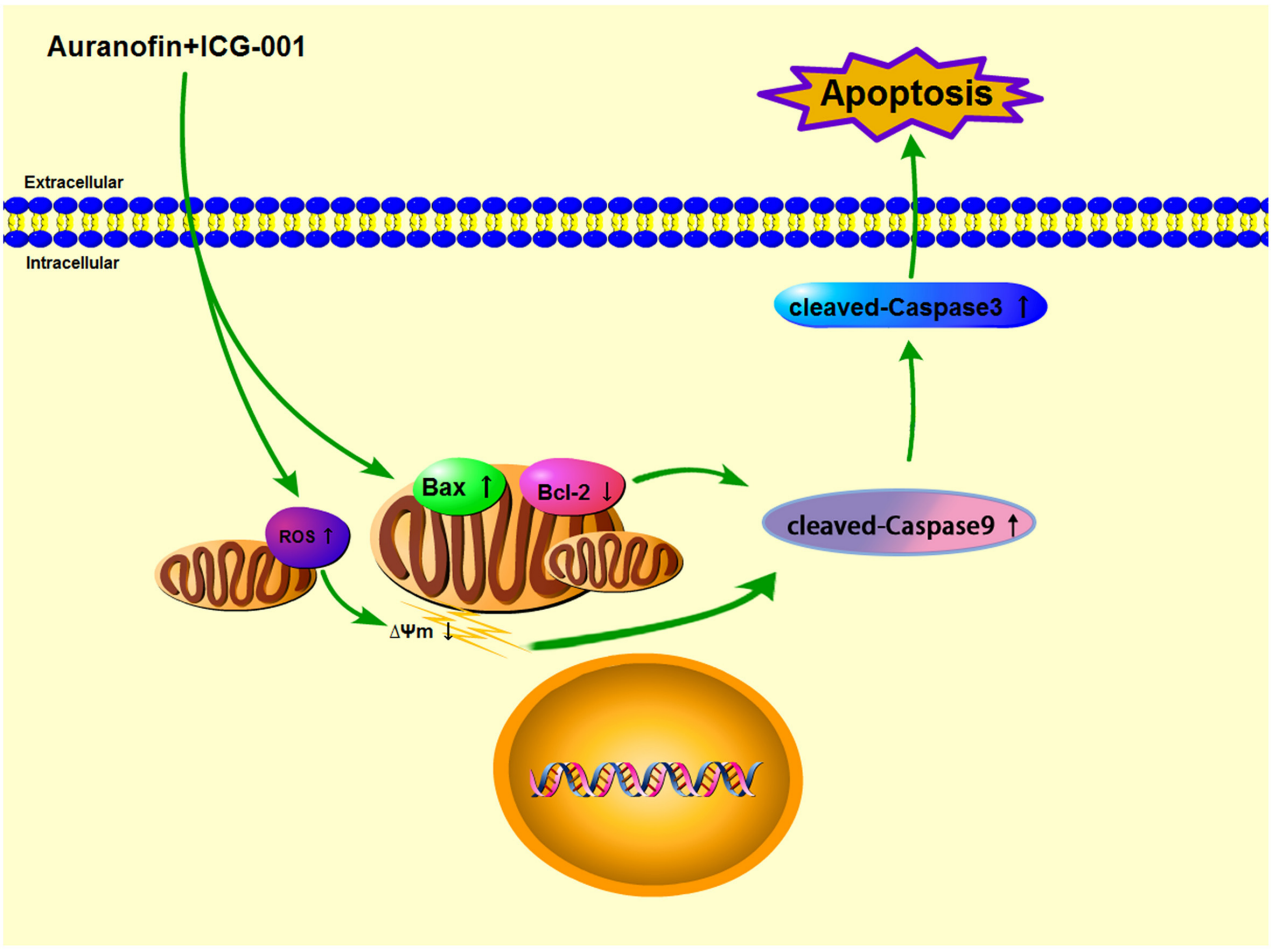
The underlying mechanism of combination of AF and ICG-001.

In conclusion, in our present research, we found that AF and ICG-001 could synergistically inhibit the proliferation of CBC *in vitro* and *in vivo* by inducing apoptosis through the mitochondrial pathway (Figure 8).

## Discussion

Combination therapy is applying multiple drugs for treating diseases and reducing suffering, and thus has been widely used and become the leading choice for treating the most dreadful diseases, such as cancer and infectious diseases (2). Due to the great benefits, it has raised extensive attention in human neoplasia researches (44–46). There are also several reports about applying combination therapy on CBC (47–49), but the effective drug combination for treating CBC is still in need.

AF has been attested by the FDA as a drug that treats human rheumatism, of which the anti-tumor effect was discovered in recent years. The enhancement of oxidative stress is frequently mentioned in the studies exploring the synergistical anti-tumor effect of AF with other drugs (25, 26). The regulation of oxidative stress is an important factor in tumor development, targeting the antioxidant capacity of tumor cells can have a positive therapeutic impact (50). ROS is a major source of oxidative stress, and many antioxidative systems of the cell keep ROS at a basal non-toxic level (i.e., redox biology), any deviation from this balance could be used for ROS signaling reactions (51). Since AF is an inhibitor of thioredoxin receptor (TrxR) (16), we assumed that the anti-tumor effect of AF is closely related with oxidative stress, which could be enhanced by other agents to achieve a synergistical anti-tumor effect. In addition, considering that AF is a commercialized drug that has already been applied in the clinic for years, this combination therapy is worth further assessment in clinical studies.

In our present study, after screening a few common chemotherapeutic drugs and novel target drugs (the data was not shown), we found ICG-001, a small molecule inhibitor of Wnt/β-catenin pathway (33), could cooperate well with AF to suppress the growth of CBC cells (Figures 1, 2). Then the transcriptome sequencing results indicated the combination therapy of AF and ICG-001 influenced redox related genes and apoptotic genes of CIPp cells (Figure 3). In addition, we further confirmed that this combination therapy could destroy the mitochondrial membrane of CIPp cells (Figure 4) and cause the loss of ΔΨm on both cell lines (Figures 5A,B). Mitochondrion is essential in cell breath and proliferation, and it has been confirmed that mitochondrion is involved in cell death (5, 10). Further, our results indicated the combination therapy of AF and ICG-001 was directly related with ROS production (Figures 5C,D). ROS is reported to be related with the loss of ΔΨm, meanwhile, ROS and ΔΨm are both related with cell apoptosis (4, 15).

Previous studies claimed that AF or ICG-001 induced cell apoptosis when they were applied alone (24, 33) or with other drugs (52, 53). In the present research, we also found the combination therapy could induce mitochondrial-mediated cell apoptosis *in vitro* and *in vivo* (Figures 6, 7B). In addition, our results showed the combination therapy triggered apoptosis through enhancing the cleaved-Caspase-9 and cleaved-Caspase-3 expression (Figure 6A). We also found the higher expression level of cleaved-Caspase-8 in the combination group than that in others (Figure 6A), therefore, the combination of AF and ICG-001 might also influence the extrinsic apoptosis pathway, and this is worth further study to confirm.

Except for participating in apoptosis, mitochondrion is also involved in autophagy (54). Interestingly, our TEM photographs showed mitochondria in autophagosome (Supplementary Figure S1), indicating that the anti-tumor effect of AF and ICG-001 might relate with mitophagy. Mitochondria can be divided into small fragments by a phagophore-mediated event to format autophagosomes, and then degraded by mitochondria-specific autophagy, i.e., mitophagy (54). It is also reported that the endoplasmic reticulum (ER)-mitochondria contact site is important in autophagosome formation (55), and the damaged or uncoupled mitochondria can be recruited by Parkin, the PARK2 gene product, to help the clearance of the organelles through the autophagosome (56). Besides, it is also reported that ICG-001 is related with cell autophagy (57). Therefore, the pathways underneath the cell death induced by AF and ICG-001 combination worth further exploration.

In conclusion, the combination of AF and ICG-001 could synergistically suppress CBC *in vitro* and *in vivo* by inducing mitochondrion damage and low ΔΨm, and producing ROS, then leading mitochondrion associated apoptosis (Figure 8). While for being applied in clinic, the underlying mechanisms of this combination therapy is worth further research.

## Data Availability Statement

The datasets presented in this study can be found in online repositories. The names of the repository/repositories and accession number(s) can be found below: https://www.ncbi.nlm.nih.gov/sra, accession ID: PRJNA763886.

## Ethics Statement

The animal study was reviewed and approved by Animal Ethics Committee of the China Agricultural University.

## Author Contributions

ZhL contributed to conceptualization, methodology, data curation, and original draft preparation. HZ organized the conceptualization, methodology, software, and review and editing of the manuscript. DL contributed to conceptualization and funding acquisition. ZiL arranged the data of this study. YZ and NC contributed to the methodology. DZ supported as project administration. JL was the supervision. All authors contributed to the article and approved the submitted version.

## Funding

ZhL, ZiL, YZ, NC, DZ, JL, and DL were funded by the National Natural Science Foundation of China (No. 31572578), and the article processing charges China Agricultural University, HZ was funded by the National Natural Science Foundation of China (No. 31902336) and the Start-up Research Grant Scheme of Hainan University [NO. KYQD (ZR) 1941].

## Conflict of Interest

The authors declare that the research was conducted in the absence of any commercial or financial relationships that could be construed as a potential conflict of interest.

## Publisher's Note

All claims expressed in this article are solely those of the authors and do not necessarily represent those of their affiliated organizations, or those of the publisher, the editors and the reviewers. Any product that may be evaluated in this article, or claim that may be made by its manufacturer, is not guaranteed or endorsed by the publisher.

## Supplementary Material

Supplementary Figure S1 Autophagosomes of the CIPp cells in combination group. Arrows: autophagosome contained mitochondrion. **(A)** 20,000 ×, bar = 1 μm. **(B)** 30,000 ×, bar = 0.5 μm.

Supplementary Figure S2 The drug toxicity assay of mice model. **(A)** The body weight of the xenograft tumor mice during drug treatment. **(B)** Histopathological examination (HE stain) of lung, liver, kidney, spleen, and heart from mice bearing with the xenograft tumor. Bar = 100 μm.
