# Supplementary figures and images for: Auranofin and ICG-001 Emerge Synergistic Anti-tumor Effect on Canine Breast Cancer by Inducing Apoptosis via Mitochondrial Pathway
Source: Front Vet Sci. 2021 Dec 15;8:772687. doi: 10.3389/fvets.2021.772687 (PMC8714754; doi:10.3389/fvets.2021.772687)

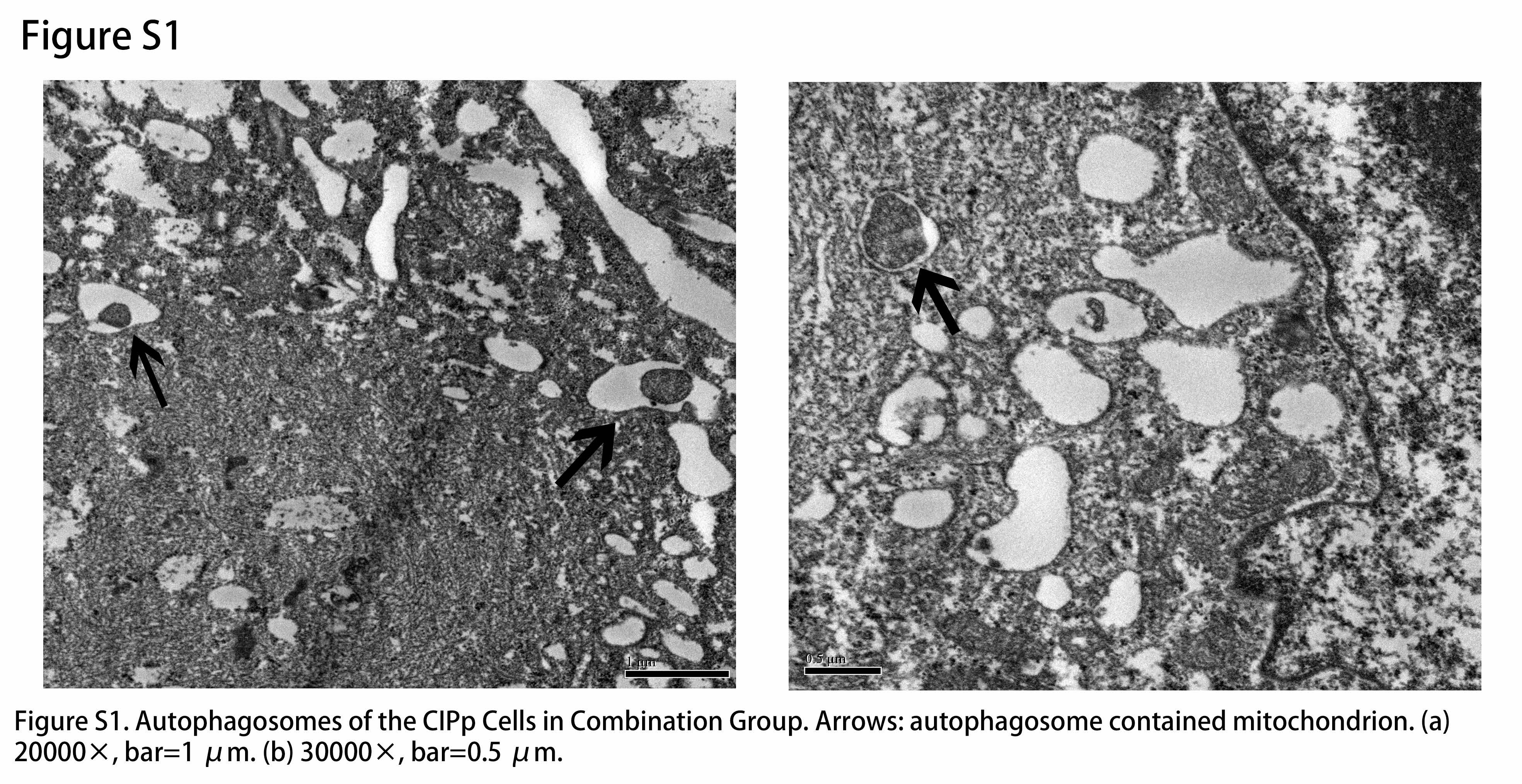

Supplement: Supplementary Figure S1 — Autophagosomes of the CIPp cells in combination group. Arrows: autophagosome contained mitochondrion. (A) 20,000 ×, bar = 1 μm. (B) 30,000 ×, bar = 0.5 μm. [file Image_1.JPEG]
